# Genetic kidney disease in adults—the pathologists’ perspective

**DOI:** 10.1093/ndt/gfag047

**Published:** 2026-03-18

**Authors:** Anna L Paterson, Melanie M Y Chan, Candice Roufosse

**Affiliations:** Department of Histopathology, Cambridge University Hospitals, Cambridge, UK; Medical Research Council Laboratory of Medical Sciences, Imperial College London, London, UK; Department of Histopathology, Imperial College Healthcare Trust, London, UK; Department of Immunology and Inflammation, Imperial College London, London, UK

**Keywords:** genetics, histopathology, kidney biopsy, monogenic

## Abstract

Monogenic diseases account for 10%–20% of chronic kidney disease (CKD) in adults. Their importance is increasingly appreciated in general nephrology, facilitated by more widespread access to genomic testing and expanding gene panels. The 2024 KDIGO clinical practice guideline for evaluation and management of CKD emphasizes the importance of considering genetic testing, particularly in the context of unexplained CKD (CKDx) when histological evaluation does not identify a precise aetiology. Identifying a genetic cause provides a definitive diagnosis and shapes patient management including access to disease-specific therapies, screening for extra-renal manifestations, early initiation of renoprotective measures, avoiding ineffective and potentially harmful treatments, a better understanding of the likely disease course including risk of recurrence following transplantation, and reproductive counselling. Despite this, genetic kidney disease may be overlooked due to lack of physician or pathologist familiarity, lack of clear family history, genotype−phenotype heterogeneity, and/or atypical presentations. The pathologist has an important role in identifying potential cases of genetic kidney disease as part of routine kidney biopsy assessment. Histology can either strengthen the case for a suspected genetic disease, or—not infrequently—be the first evidence of a potential underlying genetic cause. The pathologist participates in the clinicopathological correlation (CPC) meeting discussing evidence to identify patients in whom genetic testing would be appropriate. This review provides pathologists and nephrologists with an overview of the different histological patterns associated with genetic kidney disease, and outlines a practical framework for reporting kidney biopsies, receiving the reports, and/or participating in CPC discussions.

## INTRODUCTION

Although appreciated in the paediatric setting for many years, the prevalence and importance of monogenic causes of chronic kidney disease (CKD) in adults is increasingly recognized and estimated to account for 10%–20% of cases, with >600 kidney disease-gene associations identified [1–3].

A molecular diagnosis provides diagnostic certainty and can directly impact on patient management: access to disease-specific therapies, clinical trials, and patient support groups; screening for extra-renal manifestations; and avoidance of ineffective, potentially harmful treatments such as immunosuppression in inherited forms of focal segmental glomerulosclerosis (FSGS). Furthermore, the likely clinical course can be anticipated with early renoprotective measures, and an understanding of recurrence risk after transplantation. Finally, cascade testing of family members, screening of potential living-related donors, and reproductive counselling can be undertaken where appropriate.

**Figure 1:**
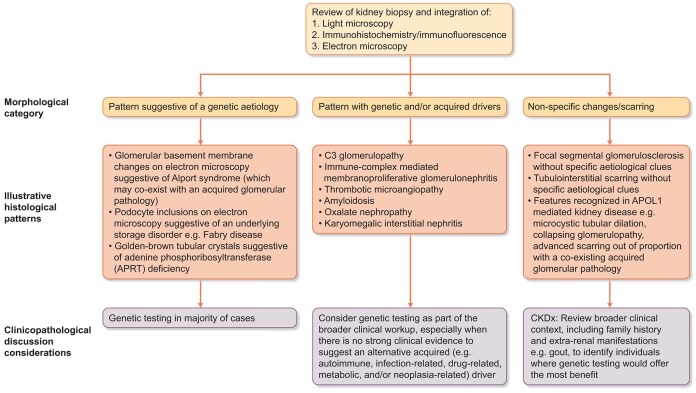
Proposed morphology-based conceptual framework when considering a monogenic aetiology for observed changes in kidney biopsies.

The initial diagnosis of monogenic kidney disease often requires clinical suspicion and may be delayed by an atypical or non-specific clinical presentation, lack of a clear family history related to variable penetrance or incomplete information, rarity of conditions, limitations of testing, and/or lack of awareness. Histopathologists have an important role in identifying cases with a potential genetic basis since the biopsy features on light microscopy and/or electron microscopy (EM) may be the first indication; therefore EM should be undertaken routinely in all biopsies whenever possible. The role of kidney biopsy assessment is to provide a morphological description and, where possible, an aetiological diagnosis (e.g. genetic, autoimmune, infection-related, drug-related, metabolic, and/or neoplasia-related), and prognostic information [4]. Categorization of monogenic kidney diseases by clinical diagnosis or genetic basis is relevant from a clinical perspective [5], but does not directly translate into specific histological appearances, as several clinical and/or genetic categories can share similar histological features. We propose a complementary morphology-based approach, starting with kidney biopsy features, with the aim of supporting the pathologist and multidisciplinary clinicopathological discussion. Using this strategy, we distinguish three broad categories, which are summarized in Fig. 1.

The first category consists of morphological patterns with a high likelihood of a genetic cause, where genetic testing is recommended. The second comprises relatively specific morphological patterns with either genetic or acquired aetiologies, where genetic testing would be appropriate in cases without strong evidence of an acquired aetiology. The third group contains non-specific chronic changes that do not suggest a particular aetiology (CKD of unexplained cause or CKDx [6]), ∼17% have an underlying genetic cause [2, 7], and the decision to undertake genetic testing is dependent on clinical context.

### Morphological patterns with high likelihood of genetic aetiology

Pathologists should be aware of characteristic features that suggest rare monogenic disorders and recommend genetic testing. These features may be the dominant abnormality or occasionally co-exist with an additional acquired process. The most frequently encountered conditions are summarized as follows.

#### Alport syndrome (AS)

AS is one of the more common monogenic kidney diseases, caused by pathogenic or likely pathogenic (P/LP) variants in *COL4A3, COL4A4*, and *COL4A5*, which can result in a phenotypic spectrum of varying severity [8]. These variants disrupt collagen IV α3α4α5 heterotrimer formation, a key structural component of the glomerular basement membrane (GBM) [9]. Presentation varies depending on the inheritance pattern, impact of the variant(s) on α3α4α5 collagen IV structure, and a complex interplay with additional factors [10].

Classical clinical and histological features of AS are seen in males with X-linked Alport syndrome (XLAS), males or females with homozygous or compound heterozygous P/LP variants of *COL4A3* or *COL4A4* (autosomal recessive Alport syndrome; ARAS), and some females with P/LP heterozygous *COL4A5* variants and presumed skewed X-inactivation. Presentation is with persistent microscopic haematuria, followed by proteinuria as GBM changes worsen and FSGS develops, and progressive CKD that usually results in kidney failure before the age of 40 in those with XLAS [11] and ARAS [12]. Individuals with heterozygous P/LP *COL4A3/A4* variants have a milder phenotype and were previously referred to as having ‘benign familial haematuria’ or ‘thin basement membrane nephropathy’. We now know that these variants are not always ‘benign’ and are associated with a ∼3% risk of developing kidney failure by the age of 80 [10]. Bilateral high frequency sensorineural hearing loss and ocular manifestations (e.g. anterior lenticonus) occur frequently in XLAS and ARAS but are not commonly seen in *COL4A3/A4* heterozygotes. While a genetics-first approach is recommended for children and young people with persistent microscopic haematuria, clinical features of AS, and/or a family history, variability in access to genetic testing means that a kidney biopsy is often performed and can be helpful to exclude alternative diagnoses such as IgA nephropathy (IgAN).

**Figure 2:**
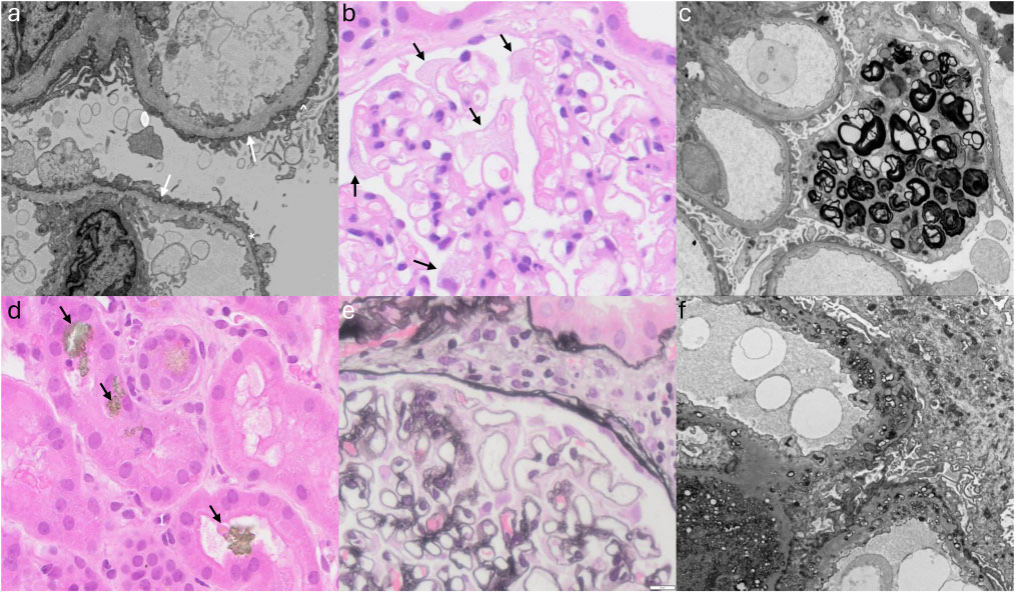
(a) Autosomal recessive AS: areas of GBM thinning (*), thickening (^), and lamellation (arrows). (b, c) Fabry disease: prominent podocytes with foamy cytoplasm (arrows) corresponding to electron dense lipid inclusions on EM. (d) Adenine phosphoribosyltransferase deficiency: brown intratubular crystals (arrows). (e, f) Lecithin-cholesterol acyltransferase deficiency: silver stain showing vacuolation of the capillary wall that corresponds to deposits identifiable on EM.

The light microscopic appearances of AS may be near normal. Over time FSGS, global glomerulosclerosis and interstitial fibrosis and tubular atrophy (IFTA) develop, often with interstitial foamy macrophages reflecting prolonged proteinuria [13]. The features are non-specific so if EM is omitted AS may be overlooked. The classically described features on EM are GBM thinning, or thickening and thinning, with lamellation (Fig. 2a) (‘basket-weave’ appearance), scalloping of the capillary loop outer surface, intramembranous lucencies, and electron dense granules [10, 13].

These classical EM features reflect one end of the Alport spectrum and typically represent the minority of cases in routine clinical practice. GBM thinning may be the only EM feature in heterozygotes. Thinning in AS may be mild, without precise thresholds that would offer high diagnostic sensitivity and specificity. Therefore, the increasing availability of genetic testing in routine clinical practice is valuable in determining which patients with more subtle EM changes of the GBM have an underlying genetic basis, allowing for final classification based on the identified genetic abnormality which will also have prognostic value. Discussions regarding the ideal nomenclature for AS more broadly are on-going [10], from the pathologists’ perspective at this time we would recommend that the histopathology report adopts a descriptive approach, noting the presence of GBM abnormalities on EM, and highlighting that this may represent an underlying monogenic disorder of GBM components such as AS. The term ‘thin basement membrane disease’ is discouraged as this suggests a specific disease entity, while in fact it is only a descriptive term with a diverse range of potential genetic and/or acquired causes.

Similar clinical presentations and/or GBM abnormalities may arise in other monogenic disorders such as those affecting podocytes (e.g. nail-patella syndrome caused by monoallelic variants in *LMX1B*, autosomal dominant *MYH9*-related disease, and biallelic variants in *MYO1E*), and other extracellular matrix GBM components (e.g. biallelic variants in *LAMB2* resulting in Pierson syndrome) [14, 15]. Comparable EM-detected structural abnormalities of Bowman’s capsule, and tubular and/or capillary basement membranes (rather than the GBM) have been described in association with monoallelic variants of *COL4A1* in hereditary angiopathy with nephropathy, aneurysm, and muscle cramps syndrome, which occasionally presents with microscopic haematuria although bilateral kidney cysts are typically the dominant clinical feature [16]. Acquired injury secondary to prolonged endothelial damage, immune-complex deposition, and/or inflammation can also result in GBM abnormalities including disruption, splitting, variable thickness, and/or thinning [13, 17, 18].

Of IgAN cases, 1.6% are reported to have thin GBMs [19], which historically was ascribed to IgAN-mediated damage [18]. More recent studies have identified *COL4A3/A4* variants in 20%–37% of IgAN patients, particularly those with GBM thickness <250 nm, GBM splitting and/or lamellation, lower serum galactose deficient-IgA1 levels, lower IgA intensity, and milder glomerular injury [19–21]. EM should be considered in cases of IgAN where the morphological features (i.e. amount of deposits, degree of hypercellularity, and scarring) do not fully explain the degree of proteinuria and/or renal dysfunction.

Genetic testing for type IV collagen variants should be considered in patients with significant ultrastructural GBM abnormalities (e.g. >50% of the GBM thinned to <250 nm in adults) or a relevant family history. However, P/LP heterozygous *COL4A3/A4* variants are seen in ∼1% of the general population [22] and are increasingly considered a risk factor for developing kidney disease, rather than disease-causing in the Mendelian sense. Therefore, clinicians should stay alert to possible co-existing glomerular pathologies [10].

#### Fabry disease

Fabry disease is an X-linked lysosomal storage disorder caused by P/LP variants in α-galactosidase A (GLA) with variants typically private to each family. Deficiency of this enzyme results in intracellular globotriaosylceramide (Gb3) accumulation in the heart, blood vessels, nerves, skin, corneas, and kidneys. Individuals typically present with cerebrovascular complications at a young age, left ventricular hypertrophy, and/or proteinuric CKD alongside acroparaesthesias (neuropathic pain), angiokeratomas, corneal opacities, hypohidrosis, and hearing loss [23]. Diagnosis is frequently delayed, particularly in males with non-classical features such as isolated renal involvement, and females who typically have an attenuated phenotype due to residual alpha-Gal-A activity [24]. Therefore Fabry disease may not be suspected prior to biopsy in these groups. Enzyme replacement is an effective treatment [25].

The classical light microscopic feature is enlarged podocytes with foamy cytoplasm reflecting lipid deposits removed during tissue processing (Fig. 2b). Mesangial expansion may be seen. The presence of secondary FSGS can result in Fabry disease being overlooked if EM is not undertaken. Vacuolation may be appreciated in distal tubular epithelial cells and arterial smooth muscle. EM reveals laminated electron dense lipid deposits in podocytes (Fig. 2c), with a concentric (myelin bodies) and/or striped (zebra bodies) pattern. Similar deposits may arise in other storage disorders and in association with drugs, notably chloroquine [26], therefore genetic assessment and/or enzymatic assessment (in males) are needed to confirm the diagnosis.

#### Adenine phosphoribosyltransferase (APRT) deficiency

APRT deficiency is an autosomal recessive disorder of adenine metabolism leading to the accumulation of 2,8-dihydroxyadenine crystals that are poorly soluble in urine. This results in a crystal nephropathy and/or radiolucent nephrolithiasis. The natural history is of recurrent kidney stones and/or urinary tract infections, progressive CKD often with accompanying proteinuria, and development of kidney failure in 20%–25%, although significant phenotypic variation exists even within families [27, 28]. A third present with acute kidney injury secondary to urinary tract obstruction [28]. The diagnosis may not be suspected in the absence of nephrolithiasis, and/or a clear family history [27–29]. Some individuals are diagnosed due to recurrence following kidney transplantation [27, 29, 30]. Treatment comprises a low purine diet, hydration, and allopurinol or febuxostat to reduce xanthine oxidase activity. Earlier treatment improves renal outcomes and if initiated prior to transplant reduces disease recurrence [27].

The characteristic biopsy finding is yellow-gold-brown needle-shaped crystals arranged in radial or irregular aggregates that birefringe under polarized light (Fig. 2d). They are found in the distal tubules, tubular epithelial cells and/or interstitium and may evoke a giant cell response [28]. Treatment can be initiated while the diagnosis is confirmed by the absence of APRT enzyme activity, urine crystal analysis, or identification of biallelic P/LP APRT gene variants.

Classical crystals may be overlooked, sparse or absent particularly in late-stage disease with extensive scarring, or misinterpreted as oxalate nephropathy, although there are some similarities in crystal architecture [27, 28, 30], their colour differs. APRT deficiency should be considered in all patients with an undetermined crystal nephropathy or nephrolithiasis.

#### Lecithin-cholesterol acyltransferase (LCAT) deficiency

LCAT deficiency is a very rare autosomal recessive disorder caused by P/LP variants in *LCAT* resulting in complete (familial LCAT deficiency) or partial (Fish-eye disease) reduction in LCAT enzyme activity. This leads to impaired HDL metabolism and accumulation of abnormal lipoprotein in tissues. Proteinuria, often nephrotic range, is the most common renal manifestation which is typically preceded by corneal opacities (which may be undiagnosed), and may be accompanied by oedema, haemolytic anaemia, thrombocytopaenia, and haematuria [31–33]. The degree of renal involvement determines prognosis, but patients typically progresses to kidney failure [33–35]. Treatment is with dietary modification.

The typical histological appearances are enlarged glomeruli with segmental PAS-positive non-argyrophilic capillary wall thickening and vacuolation, spiculations resembling membranous glomerulopathy, double contour formation at later stages, and foamy mesangial expansion (Fig. 2e) [31–34]. EM shows electron dense and electron lucent inclusions in subepithelial, intramembranous, subendothelial and mesangial areas (Fig. 2f) [31–35].

Similar although less severe EM features are seen in acquired hepatic glomerulopathy secondary to liver failure and in Alagille syndrome. Rare cases of acquired LCAT deficiency due to autoantibodies are recognized, typically in older patients, and can be accompanied by membranous glomerulopathy [36].

### Morphological patterns where a genetic aetiology is possible; genetic testing likely to be considered in clinical workup in the absence of a clear acquired driver

The second group are relatively common morphological patterns in CKD, that may each be driven by monogenic and/or acquired causes (Fig. 1). Therefore in the absence of a clear acquired driver, an underlying genetic cause should be sought. It is beyond the scope of this review to discuss each disorder in depth, key points are considered next.

#### C3 glomerulopathy (C3G) and immune-complex mediated membranoproliferative glomerulonephritis (IC-MPGN)

C3G comprises complement-mediated C3 glomerulonephritis and dense deposit disease (differentiated by EM features). These rare disorders are characterized by alternative complement pathway activation and usually present with nephrotic syndrome and progressive proteinuric CKD. Overactivation can be triggered by autoantibodies, such as C3 nephritic factor (C3Nef) which stabilizes C3 convertase, or less commonly be associated with rare genetic variants in alternative pathway complement components, e.g. *C3, CFH*, and *CFHR5* [37]. Occasionally genetic variants and acquired autoantibodies may co-exist [38]. In many cases no driver is identified [38, 39]. Genetic testing is recommended for young-onset C3G, if there is a family history of MPGN or CKDx, and/or renal transplantation or complement inhibition is being considered.

Those diagnosed with IC-MPGN require investigation for an underlying driver which may be infectious, autoimmune (systemic or drug-induced) or a haematological malignancy including a monoclonal gammopathy and/or cryoglobulinaemia. In the absence of an identified driver IC-MPGN is considered idiopathic. Studies have shown likely overlap in the pathogenesis of IC-MPGN and C3G, and sequential biopsies may show switching between these two histological patterns [39]. Until recently treatment of C3G/IC-MPGN consisted of steroids and/or mycophenolate mofetil, with limited efficacy, however recent Phase 3 data have shown promising results for complement inhibitors targeting C3 and Factor B.

#### Thrombotic microangiopathy (TMA)

TMA arises due to endothelial injury of diverse aetiologies, including ADAMTS13-mediated (thrombotic thrombocytopenic purpura/TTP), complement-mediated (atypical haemolytic uraemic syndrome/aHUS), infection, drugs, hypertensive emergencies, pregnancy, autoimmune disorders, malignancy, and transplantation. Disease may be systemic or renal-limited [40]. The histological findings are similar regardless of the driver and clinical correlation is required to identify the most likely cause(s). P/LP variants affecting the alternative complement pathway are identified in 50%–60% of aHUS cases (e.g. *CFH, C3, CFB, CFI, CFHR3-CFHR1, MCP*) although are often incompletely penetrant requiring a trigger such as infection, surgery including kidney transplantation, or pregnancy to unmask underlying complement dysregulation [37, 40]. Other genetic causes of TMA include coagulation cascade abnormalities (*DGKE, PLG, THBD*) and occasionally cobalamin deficiency (*MMACHC*) [40].

#### Amyloidosis

In Western populations ∼80% of amyloidosis is AL type secondary to monoclonal immunoglobulin deposition, 7%–14% AA type due to chronic inflammation, and less than 5% hereditary due to amyloidogenic mutations in a range of proteins including fibrinogen A α-Chain (AFib), lysozyme (ALys), gelsolin (AGel), transthyretin (ATTR), and apolipoprotein (AApo) AI, AII, and CII, with some preferentially affecting the kidney [41]. While hereditary amyloidosis is of autosomal dominant inheritance, penetrance is variable, and most individuals do not report a family history [42, 43]. Genetic testing should be considered in patients with features suggestive of hereditary transthyretin amyloidosis including restrictive cardiomyopathy, autonomic or peripheral neuropathy, proteinuria, or gastrointestinal symptoms.

**Figure 3:**
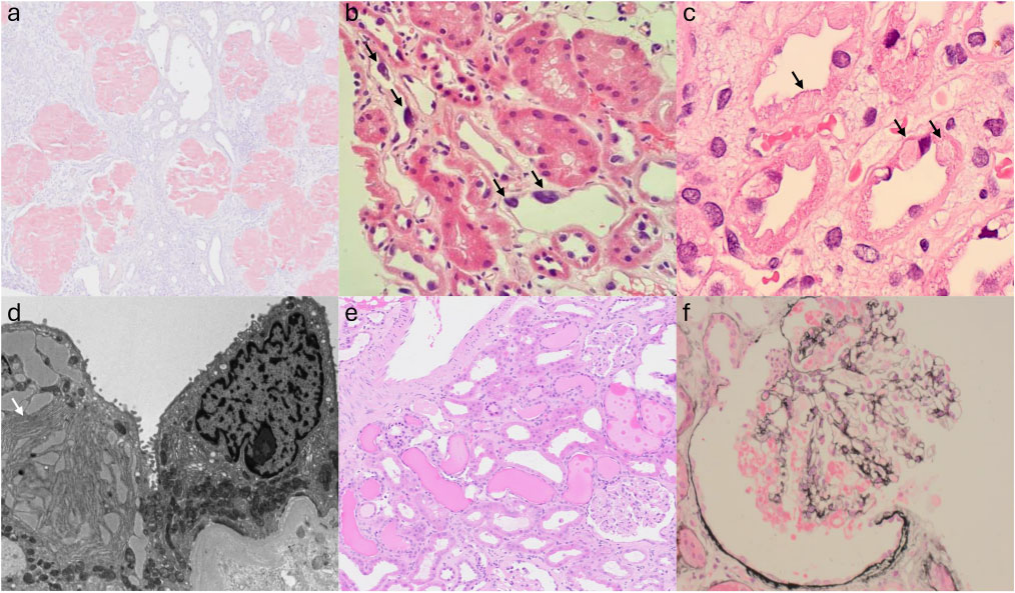
(a) Afib amyloidosis: Congo red stain showing extensive glomerular amyloid deposition. (b) Karyomegalic tubulointerstitial nephritis: karyomegalic nuclear changes (arrows) in a confirmed case with FAN1 mutation. (c, d) Autosomal dominant tubulointerstitial kidney disease-UMOD: intracytoplasmic inclusions in tubular epithelial cells (arrows), which correspond to stacks and whorls of endoplasmic reticulum visible on EM (arrow). (e, f) *APOL1* mediated kidney disease: microcystic tubular changes and a silver stain showing segmental glomerular collapse, features that would warrant consideration of background APOL1-mediated kidney disease.

In the kidney biopsy, hereditary amyloidoses divide into two broad groups: those with predominantly glomerular involvement which therefore present with significant proteinuria (fibrinogen A α, ApoAII, ApoCII, and gelsolin), and those which classically have predominantly tubulointerstitial and/or vascular involvement thus presenting with progressive CKD and mild proteinuria (ApoAI, lysozyme, transthyretin, and ApoCIII) [42]. In AFib amyloidosis, the most common hereditary amyloidosis, glomerular amyloid deposition shows widespread obliteration of capillary loops (‘cotton ball’ appearance) with minimal interstitial or vascular deposition (Fig. 3a) [42].

Immunohistochemistry and/or proteomics is required to subtype the amyloid. It is important for a pathologist to be aware of genetic causes of amyloidosis as the prognosis is better, and treatments differ from acquired amyloidosis. A sizeable proportion of patients with hereditary amyloidosis have a co-existing serum monoclonal immunoglobulin and mislabelling as AL amyloidosis may result in patients receiving inappropriate chemotherapy [42, 44].

#### Oxalate nephropathy

Oxalate nephropathy is relatively common, reported to occur in 1% of kidney biopsies [45]. Most cases are acquired due to increased intake (e.g. high oxalate foods or oxalate precursors such as ethylene glycol or vitamin C) or enteric hyperoxaluria associated with fat malabsorption resulting from chronic pancreatitis, gastric bypass surgery, or bowel resection [45]. Primary hyperoxaluria (PH) is a rare autosomal recessive disorder caused by P/LP variants in *AGXT* (PH1), *GRHPR* (PH2), or *HOGA1* (PH3), leading to hepatic overproduction of oxalate with systemic deposition in multiple organs including the kidneys. PH1 accounts for 80% of cases and presents with recurrent calcium oxalate kidney stones and nephrocalcinosis, with 50% developing kidney failure by young adulthood if untreated [46].

Both primary and acquired forms have similar histological features of intratubular birefringent pale crystals with additional accumulation in tubular epithelial cells and/or the interstitium. There is typically acute tubular injury and if deposition is chronic, IFTA develops that may have accompanying interstitial inflammation without tubulitis [45]. In the absence of an acquired cause, a 24-h urine collection for oxalate should be performed, with hyperoxaluria prompting genetic testing for PH.

Treatment with RNA interference (RNAi) therapy (lumasiran/nedosiran) reduces urinary oxalate excretion in patients with PH1 and is recommended as first-line alongside pyridoxine supplementation [47, 48]. In those with kidney failure but without access to RNAi therapy, combined liver-kidney transplant should be considered to reduce recurrence risk and premature graft failure [49].

#### Karyomegalic interstitial nephritis

Karyomegalic interstitial nephritis is characterized by enlarged, often hyperchromatic nuclei in tubular epithelial cells (Fig. 3b). It may be an acquired change reflecting cytopathy due to viral infection, drugs such as ifosfamide, toxins including ochratoxin that may contaminate food, or heavy metals. In some cases, and particularly if the features are widespread, it may represent systemic karyomegaly, a very rare autosomal recessive disorder caused by variants in DNA repair gene *FAN1* [50]. The clinical course is of slowly progressive CKD often leading to kidney failure before the age of 50, and is associated with recurrent upper respiratory tract infections, cholestatic liver function tests, and a possible increased malignancy risk [51]. There are currently no specific treatments.

### Non-specific morphological patterns which may have a genetic aetiology

The third group is common, as it comprises FSGS where immunohistology and EM have not identified a specific cause, and IFTA without histological aetiological clues. Therefore, even after biopsy the CKD remains unexplained (CKDx) [6]. A proportion will have an underlying monogenic cause or risk factor, which is not associated with specific morphological features. Clinicopathological review is important to determine which patients would most benefit from genetic assessment.

#### Focal segmental glomerulosclerosis

FSGS is characterized by podocyte injury and depletion. It has diverse aetiologies classified into primary, genetic, secondary, and FSGS of undetermined cause [52]. Around 60 monogenic causes of FSGS have been reported and account for ∼10%–20% of adult-onset FSGS, with variants in *COL4A3/A4/A5* identified most frequently [53, 54].

Autosomal dominant forms of FSGS, associated with variants in *ACTN4, INF2*, and *TRPC6* [55–57], present later than autosomal recessive forms and often do not manifest the full-blown nephrotic syndrome. The common hypomorphic *NPHS2* R229Q allele is also associated with late-onset FSGS when inherited *in trans* with a second *NPHS2* P/LP variant [58]. A FSGS case series reported a diagnostic yield for genetic causes of 88% in cases with a clinicopathological mismatch (nephrotic syndrome with <80% podocyte foot process effacement (PFPE) or >80% PFPE without nephrotic syndrome) and 62% in secondary FSGS (no nephrotic syndrome and <80% PFPE) of undetermined cause [52]. A monogenic cause was rare (8%) in primary FSGS (nephrotic syndrome and >80% PFPE) [52]. Genetic testing should be considered for patients with steroid-resistant nephrotic syndrome, FSGS of undetermined cause, and/or a family history where the result may impact management, either through avoidance of immunosuppression or to allow kidney donation from an unaffected relative.

#### Autosomal dominant tubulointerstitial kidney disease (ADTKD)

ADTKD in adults is most commonly due to P/LP variants in *UMOD* or *MUC1* and characterized by a bland urinary sediment, gout, and slowly progressive CKD leading to kidney failure in the fourth to fifth decade [59, 60]. Kidney biopsy shows non-specific IFTA, tubular microcysts in some cases, tubular basement membrane thickening that may be lamellated, and/or patchy interstitial inflammation. ADTKD-*UMOD* may be suspected based on intracytoplasmic inclusions in the tubular epithelial cells of the loop of Henle, visible on H&E, PAS, or trichrome stains, which correspond to stacks and whorls of endoplasmic reticulum on EM (Fig. 3c,d). The glomerular compartment is typically spared although FSGS is occasionally present. This may result in a family’s phenotype being misclassified as FSGS/hereditary glomerular disease and a negative genetic result if only a glomerular-orientated panel is undertaken. In patients with an initial negative genetic result but high clinical suspicion, additional testing should be undertaken to identify frameshift variants in the GC-rich coding variable number tandem repeat (VNTR) domain of *MUC1* that are difficult to detect using short-read sequencing. Options include allele-specific probe extension assays (e.g. SNaPshot minisequencing), long-read sequencing, computational tools to identify *MUC1* VNTR variants from short-read sequencing (VNtyper), and immunohistochemistry to detect the frameshift mucin-1 protein (MUC1fs) in kidney tissue [61–64].

#### Nephronophthisis (NPHP)

NPHP is a rare, autosomal recessive primary ciliopathy that results from biallelic P/LP variants in >20 different genes, which impair functioning of the primary cilia, basal bodies, and centrosomes. Most adult/late-onset forms are due to biallelic P/LP variants in *NPHP1*, with homozygous large gene deletions seen frequently [65]. Individuals have bland urine, impaired urinary concentrating ability, and corticomedullary cyst formation. CKD typically progresses to kidney failure during childhood or adolescence [65, 66]. Extra-renal manifestations are seen in ∼20% and vary by gene affected, including retinitis pigmentosa, skeletal defects, congenital hepatic fibrosis, and cerebellar hypoplasia and ataxia [65]. In cases where the kidney is biopsied, almost all show non-specific IFTA with other features being cortical microcysts, GBM abnormalities, and/or tubular membrane thickening with lamellation [65, 67], none of these features being specific for NPHP.

#### *APOL1* mediated kidney disease (AMKD)

Biallelic gain-of-function *APOL1* risk variants (G1: two linked missense variants and G2: six base pair deletion) are associated with an increased risk of proteinuric CKD in individuals with recent African ancestry [68, 69]. Individuals with two high-risk *APOL1* variants have 25% higher risk of CKD and 84% higher odds of FSGS than low-risk carriers [70], but only ∼20% develop kidney disease, usually in association with an interferon-driven second hit [71]. Access to *APOL1* genotyping is currently variable and should be undertaken with appropriate counselling. Recognition of AMKD may influence surveillance, management, and allow clinical trial enrolment [72]. In the context of transplantation, the increased CKD risk travels with the donated kidney therefore grafts from donors with two high-risk *APOL1* variants may have an increased risk of collapsing glomerulopathy and shorter survival [73].

From a pathologist’s perspective, AMKD has no specific diagnostic features, and at the same time, diagnostic features of other diseases (e.g. lupus nephritis) can be present as triggers [73–75]. However certain changes are more frequent and may warrant the possibility of AMKD being raised, in the context of clinical information on patient ancestry. These include microcystic tubular dilation to 2–3 times normal typically with intraluminal PAS-positive casts and epithelial flattening (Fig. 3e) [74–76], and collapsing glomerulopathy (Fig. 3f). More chronic injury also tends to be observed in individuals with a glomerulopathy on a background of AMKD [74, 75].

## SUMMARY

To conclude, nephropathologists should be aware of the full spectrum of genetic causes of kidney disease and potential presentations. We advocate considering the possibility of a genetic basis as routine to reduce risk of oversight. In many cases, a genetic aetiology can rapidly be discounted, but in those where a genetic cause is possible, this should be conveyed in the histopathology conclusion. This review provides a framework for clinicopathological reasoning and highlights the importance of routine EM to allow identification of diagnostic features that may not be anticipated from light microscopy. Discussion of clinical context alongside histological features guides which patients would benefit from genetic evaluation. Results of subsequent genetic testing should be relayed to histopathologists as well as the nephrologists, to refine final disease classification.

## Data Availability

Not applicable.
